# Gait speed and individual characteristics are related to specific gait metrics in neurotypical adults

**DOI:** 10.1038/s41598-023-35317-y

**Published:** 2023-05-18

**Authors:** Maryana Bonilla Yanez, Sarah A. Kettlety, James M. Finley, Nicolas Schweighofer, Kristan A. Leech

**Affiliations:** 1 Division of Biokinesiology and Physical Therapy, University of Southern California, Los Angeles, CA USA; 2 Neuroscience Graduate Program, University of Southern California, Los Angeles, CA USA

**Keywords:** Rehabilitation, Neurological disorders

## Abstract

Gait biofeedback is a well-studied strategy to reduce gait impairments such as propulsion deficits or asymmetric step lengths. With biofeedback, participants alter their walking to reach the desired magnitude of a specific parameter (the biofeedback target) with each step. Biofeedback of anterior ground reaction force and step length is commonly used in post-stroke gait training as these variables are associated with self-selected gait speed, fall risk, and the energy cost of walking. However, biofeedback targets are often set as a function of an individual’s baseline walking pattern, which may not reflect the ideal magnitude of that gait parameter. Here we developed prediction models based on speed, leg length, mass, sex, and age to predict anterior ground reaction force and step length of neurotypical adults as a possible method for personalized biofeedback. Prediction of these values on an independent dataset demonstrated strong agreement with actual values, indicating that neurotypical anterior ground reaction forces can be estimated from an individual’s leg length, mass, and gait speed, and step lengths can be estimated from individual’s leg length, mass, age, sex, and gait speed. Unlike approaches that rely on an individual’s baseline gait, this approach provides a standardized method to personalize gait biofeedback targets based on the walking patterns exhibited by neurotypical individuals with similar characteristics walking at similar speeds without the risk of over- or underestimating the ideal values that could limit feedback-mediated reductions in gait impairments.

## Introduction

Gait training is a common component of rehabilitation paradigms for individuals with gait dysfunction secondary to musculoskeletal and neurological conditions. This training typically involves interventions that target stability, endurance, speed, and movement patterns during walking^1–5^. In this context, clinicians often provide external cues or feedback to change different aspects of walking that are associated with gait speed^6,7^, fall risk^8–10^, orthopedic injury^11,12^, and metabolic cost^6,10,13,14^ in many patient populations. This has prompted research studies designed to evaluate the use of real-time biofeedback to change gait biomechanics.

These studies have largely focused on the effects of visual gait biofeedback, with kinematic and kinetic gait variables most often fed back to the learners^15^. In studies of gait dysfunction post-stroke, for example, visual biofeedback is often used to promote increases in step length (and reductions in step length asymmetry)^16–19^ or increases in peak anterior ground reaction force^20,21^. These gait biofeedback variables are also common targets in studies of gait in older adults^22,23^ and individuals with Parkinson’s Disease^24,25^.

However, recent literature reviews have highlighted that the results across studies of gait biofeedback are inconsistent (for review see van Gelder, et al. 2018^15^ and Spencer, et al. 2021^26^); due, in part, to methodological heterogeneity. They found that studies differ in the modality of the biofeedback provided and the gait variable fed back to the learner. Even between studies that are matched for these factors, the methods to set the target values for biofeedback schemes vary. Step length biofeedback targets for people post-stroke have been set in a variety of ways. Some studies ask participants to lengthen their shorter step to the length of their longer step^18,19^, while others instruct participants to make the right and left step lengths equal by setting the target to be the average of the two step lengths^27^ or without any direction on which step length they should change^28,29^. Anterior ground reaction force biofeedback targets are often set as a percent increase from baseline walking values, but the magnitude of this increase varies between studies^22,30^.

The methodological variability in biofeedback target setting reflects our limited understanding of the desired target magnitudes for these metrics. While there seems to be broad agreement that the targets should be individualized, this is currently achieved by setting biofeedback targets as a function of an individual’s baseline walking pattern. However, this may over- or underestimate the true desired target values for these metrics, and there is currently no way to determine a priori if this method of target setting is appropriate or accurate. One viable approach may be to understand the magnitudes of step length and anterior ground reaction force exhibited by neurotypical individuals of similar ages and body morphologies, walking at similar speeds.

Here, we built prediction models for peak anterior ground reaction force and step length of neurotypical adults using individual characteristics and gait speed. Then, we evaluated if these models could be used to predict the anterior ground reaction forces and step lengths of neurotypical adults in an independent dataset. We also compare the performance of this prediction model to the performance of age- and sex-based norms reported for peak anterior ground reaction force^31^ and step length^32^. We hypothesized that predicting peak anterior ground reaction force and step length using our prediction equations (that include leg length, mass, age, sex, and gait speed) would allow for an accurate individualized estimation of these gait metrics beyond that provided by age- and sex-based norms. This work will provide the framework for a more standard methodology to personalize biofeedback targets in gait rehabilitation research.

## Results

In this study, we completed a secondary analysis of two previously collected gait datasets to create models based on speed, leg length, mass, sex, and age to predict peak anterior ground reaction force and step length of neurotypical adults. These models were then validated on an independent dataset and compared to the accuracy of normative values for those individuals. Speed was included in the models given its known relationship with peak anterior ground reaction force and step length^33^. Leg length was included as a proxy for height, given that step length scales with height^34^ and height was not reported in the independent dataset. Mass was included because ground reaction forces scale with mass^34^. Biological sex was included secondary to normative data that suggests an effect of sex on step lengths^32^. Age was also included given the evidence that step length and anterior ground reaction forces change with age^35–38^. One dataset was used to train the models (a training dataset) and the other to validate them (an independent validation dataset).

### Model selection

Best subset model selection was used to identify the best models for the training dataset. The final step of the model selection process for peak anterior ground reaction force is shown in Table 1 and for step length in Table 2. The model with the lowest Akaike information criterion (AIC) was selected as the final prediction model for each variable.

**Table 1:** Best subset selection for peak anterior ground reaction force model.

Model	AIC
AGRF ~ Speed + (1 | ID)	2473.0
AGRF ~ Speed + (Speed | ID)	2264.8
AGRF ~ Speed + Speed_2 + (Speed | ID)	2209.7
AGRF ~ Speed + Mass + Speed_2 + (Speed | ID)	2210.2
AGRF ~ Speed + Leg length + Mass + Speed_2 + (Speed | ID)*	2202.1
AGRF ~ Speed + Sex + Leg length + Mass + Speed_2 + (Speed | ID)	2203.1
AGRF ~ Speed + Age + Sex + Leg length + mass + Speed_2 + (Speed | ID)	2204.1

The last step of the best subset selection process is shown. Comparisons across models of an increasing number of variables. The final model chosen had the lowest AIC, highlighted with (*).

*AGRF* anterior ground reaction force, *ID* participant identification, *SPEED_2* speed-squared.

**Table 2:** Best subset selection for step length model.

Model	AIC
SL ~ Speed + (1 | ID)	− 1153.7
SL ~ Speed + (Speed | ID)	− 1226.7
SL ~ Speed + Speed_2 + (Speed | ID)	− 1333.6
SL ~ Speed + Leg length + speed_2 + (Speed | ID)	− 1336.2
SL ~ Speed + Mass + Leg length + Speed_2 + (Speed | ID)	− 1336.6
SL ~ Speed + Sex + Mass + Leg length + Speed_2 + (Speed | ID)	− 1334.9
SL ~ Speed + Age + Sex + Mass + Leg length + Speed_2 + (Speed | ID)*	− 1339.7

The last step of the best subset selection process is shown. Comparisons across models of an increasing number of variables. The final model chosen had the lowest AIC, highlighted with (*).

*SL* step length, *ID* participant identification, *SPEED_2* speed-squared.

### Prediction of peak anterior ground reaction force magnitude

**Figure 1:**
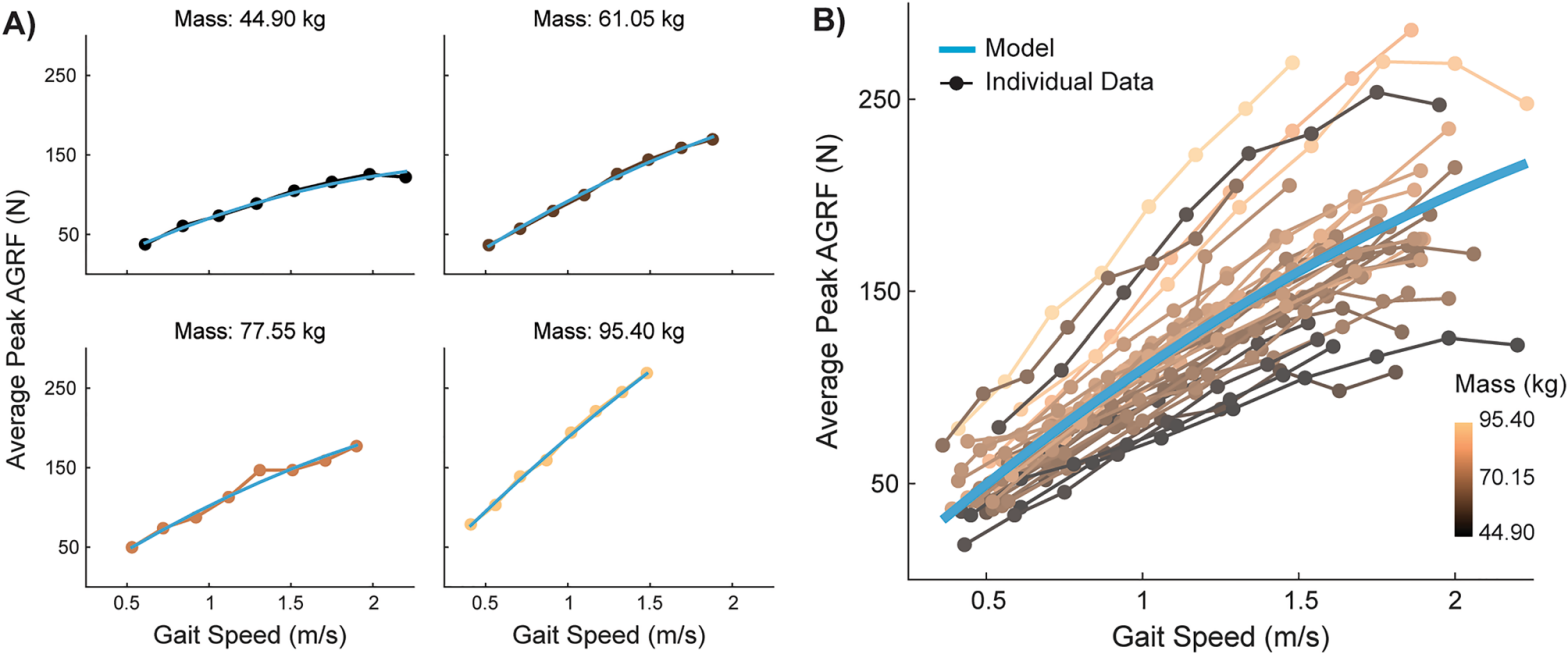
Model of peak anterior ground reaction force displayed against individual data. (**A**) Representative individual model fits with fixed and random effects of data from participants of different masses. Since we included a random slope for speed and a random intercept, the individual model fits varied in steepness and intercept, depending on the participant. (**B**) Mean fixed effects model of peak anterior ground reaction force (blue line) plotted with all individual data. This model was visualized through simulated fixed effect values based on the original data. Specifically, we created a vector of 20 speeds ranging from the minimum to the maximum speeds and found the age, mass, and leg length group means. These simulated values were used with the fixed-effects only model (for men, as sex was coded in the model as male = 0) to calculate predicted values that are plotted in blue. Individual data are plotted on a color spectrum from light orange to dark brown to represent participant mass as a third dimension of the data.

The model that best predicted peak anterior ground reaction force (AGRF) magnitude included fixed effects for speed, speed^2^, mass, and leg length, as well as a random slope (speed) and intercept. Figure 1A displays examples of individual model fits for the training data. Figure 1B shows the mean fixed-effects model displayed against individual training data. The non-standardized and standardized beta weights for each predictor in the peak anterior ground reaction force model are provided in Table 3. The standardized beta weights indicate that the primary predictor of peak anterior ground reaction force in neurotypical gait is gait speed.

**Table 3:** Non-standardized betas and standardized beta weights for the peak anterior ground reaction force model.

Independent variable	Non-standardized beta	Standardized beta
Speed	149	1.24
Speed_2	− 19.2	− 0.382
Leg length	− 127	− 0.155
Mass	0.695	0.151
Intercept	32.2	0.0106

The coefficients for each independent variable (and intercept) in the model are displayed in order of the absolute magnitude of the standardized beta weight.

**Figure 2:**
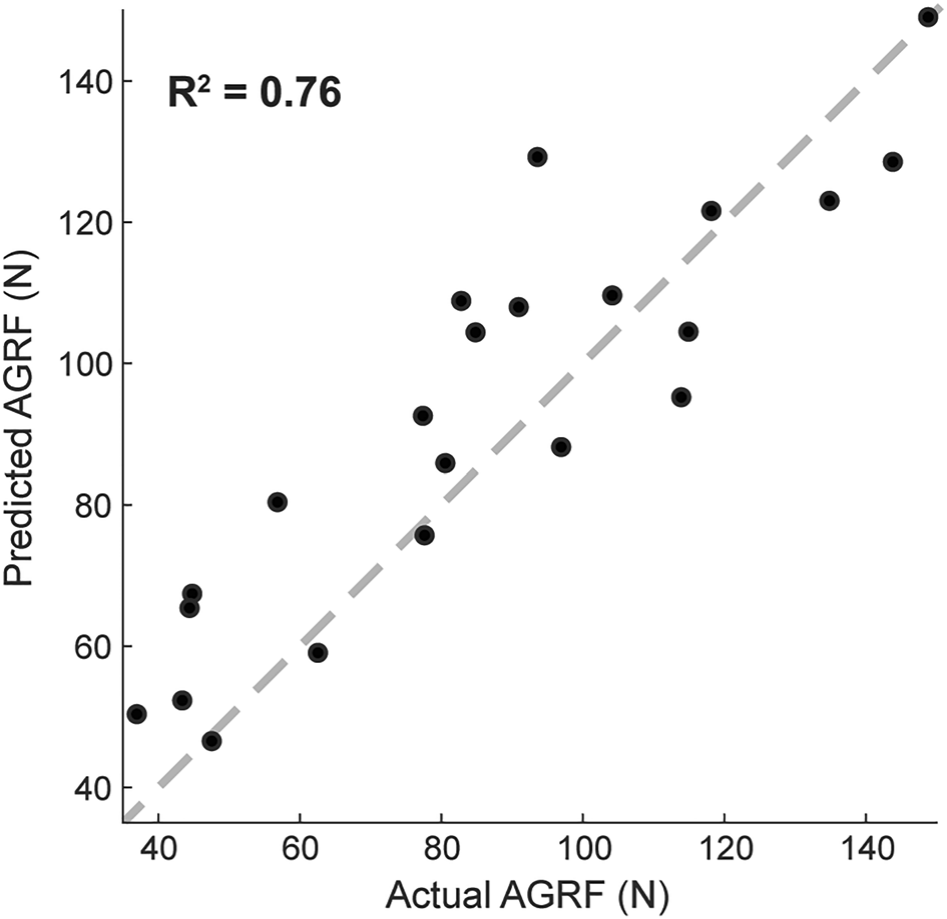
Testing the peak anterior ground reaction force (AGRF) prediction model on an independent data set. Values predicted by the model were in strong agreement with the actual values of peak anterior ground reaction force from the independent data set. The unity line is displayed as the gray dashed line.

Predictions from this model of peak anterior ground reaction force showed strong agreement with the actual values of peak anterior ground reaction force magnitude in an independent dataset (N = 22; Fig. 2; R^2^ relative to the unity line = 0.76). These predictions had an RMSE = 15.98 N. Furthermore, relative to the actual values of peak anterior ground reaction force, predictions from this model had a significantly smaller mean absolute error (2.1 ± 1.8) than normative values (7.2 ± 4.2; p = 7.00E-6).

### Prediction of step length magnitude

**Figure 3:**
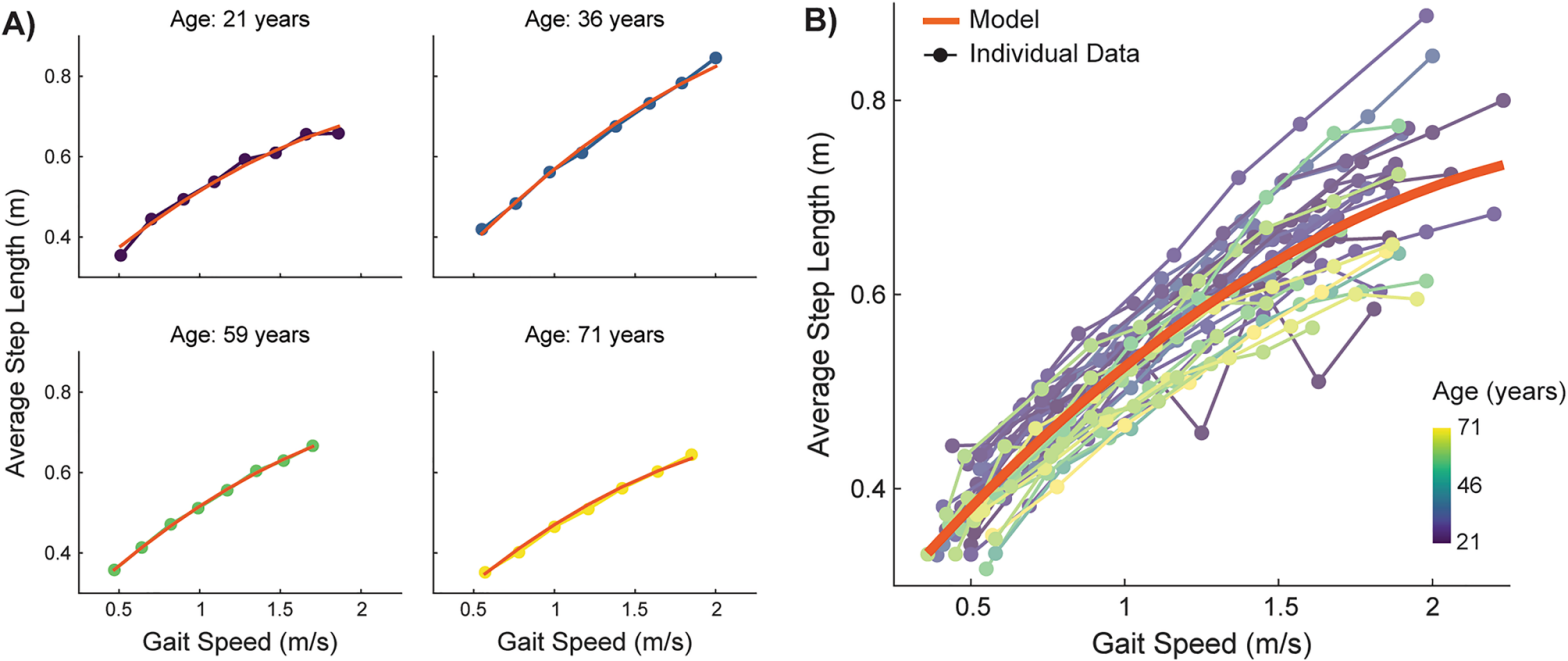
Step length model displayed against individual step length data. (**A**) Representative individual model fits with fixed and random effects across the age spectrum. Since we included a random effects term for speed and a random intercept, the individual models varied in steepness and intercept, depending on the participant. (**B**) Mean fixed effects model of step length (orange line) plotted with all individual data. This model was visualized with the same methods described in Fig. 1B. Individual data are plotted in colors ranging from yellow to green to blue to represent each participant’s age.

The model that included fixed effects for speed, speed^2^, age, mass, sex, and leg length as well as a random slope (speed) and intercept best-predicted step length (SL) magnitude. Example individual model fits on training data are displayed in Fig. 3A, and the mean fixed-effects model against all individual training data are in Fig. 3B. The non-standardized and standardized beta weights for each predictor in the step length model are provided in Table 4. The standardized beta weights of this model indicate that the primary predictor of neurotypical step length is gait speed.

**Table 4:** Non-standardized betas and standardized beta weights for the step length model.

Independent Variable	Non-standardized Beta	Standardized Beta
Speed	0.395	1.55
Speed_2	− 0.0701	− 0.660
Age	− 6.92E−4	− 0.103
Leg length	0.127	0.0733
Mass	6.04E−4	0.0618
Sex	− 1.26E−3	− 0.0111
Intercept	0.0881	− 0.005

The coefficients for each independent variable (and intercept) in the model are displayed in order of the absolute magnitude of the standardized beta weight.

**Figure 4:**
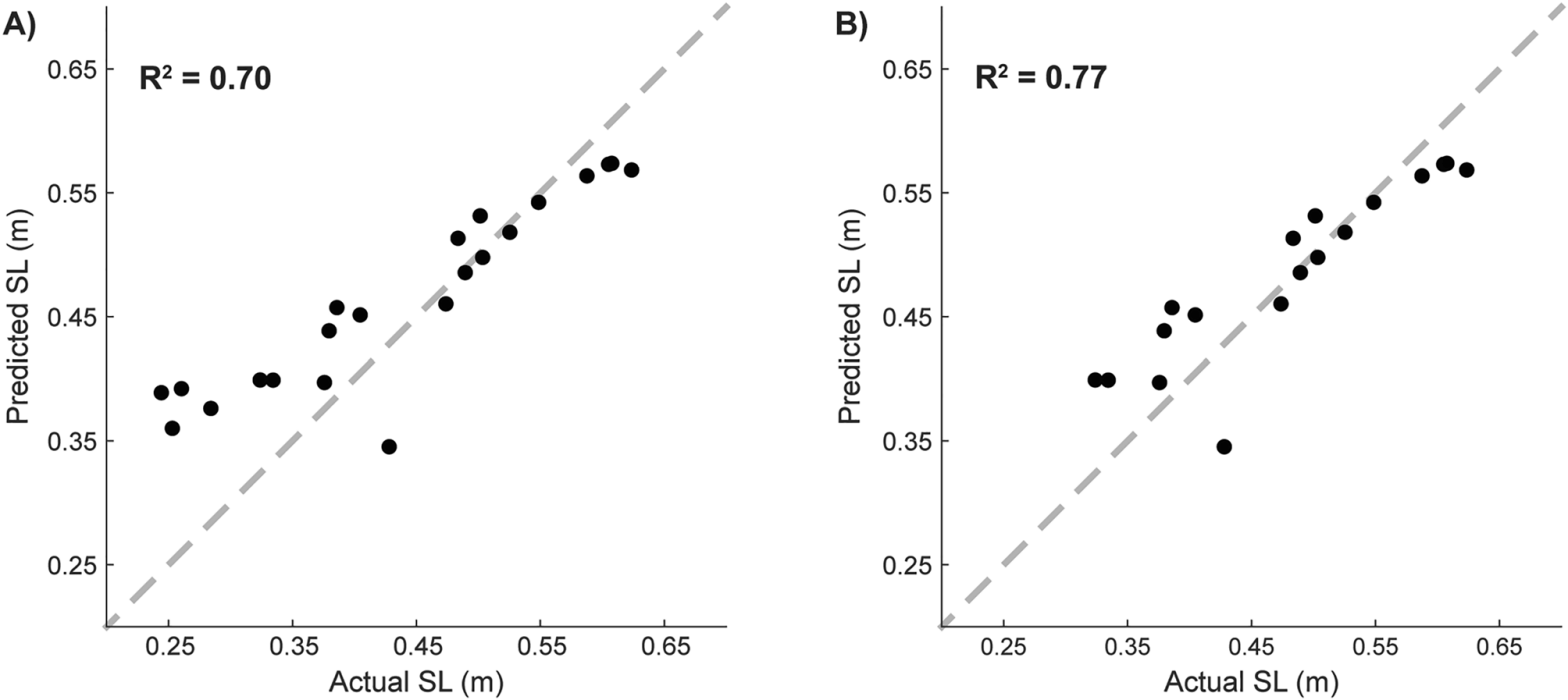
Testing the step length (SL) prediction model on an independent data set. (**A**) Predicted values from the model were in moderate agreement with the actual step length magnitudes of the entire independent data set (N = 23). (**B**) The agreement between the predicted and actual step length values improved when we predicted only the step length values that were within the range of the step lengths represented in the training data set (N = 18). The gray dashed line in both plots represents the unity line.

Predictions from this model of step length showed moderate agreement with the actual values of step length magnitude within the full independent dataset (N = 22; Fig. 4a; (R^2^ relative to the unity line = 0.70). These predictions had an RMSE = 0.07 m. Of note, the model overestimated the actual step lengths that were < 0.31 m, which were not represented in the training dataset.

Because five of the participants within the independent validation set exhibited step lengths that were below the range of step lengths in the training set, we performed the same analysis on a subset of the participants who exhibited step lengths within the range of step lengths in the training set (0.31–0.85 m; N = 18). Step length predictions for this subset of participants showed strong agreement with the actual values (N = 18; Fig. 4b; R^2^ relative to the unity line = 0.77) and had a smaller RMSE of 0.04 m. Relative to the actual values of step length, predictions from this model had a significantly smaller mean absolute error (0.04 ± 0.03) than normative values (0.13 ± 0.08; p = 9.23E−5).

Spreadsheets to calculate the predicted magnitudes of peak anterior ground reaction force and step length for an individual are provided in the Supplementary Material S1.

## Discussion

In this study, we developed models to predict the magnitudes of peak anterior ground reaction force and step length observed in neurotypical individuals while walking at different speeds. Both models accounted for the individual’s gait speed, mass, and leg length, while the prediction model for step length also included terms for age and sex. We then tested the performance of these prediction models on an independent dataset. The model-predicted magnitudes of peak anterior ground reaction force and step length were in strong and moderate agreement with the actual values of these measures, respectively. Moreover, the model-predicted magnitudes for both parameters were more accurate than age- and sex-based norms (demonstrated by smaller mean absolute errors). This suggests that these prediction models can generate reasonable estimates of individualized reference values for peak anterior ground reaction force and step length during walking at a wide range of gait speeds. This has important implications for gait rehabilitation research or clinical gait training—the model equations can be used to calculate peak anterior ground reaction force and step length magnitudes that reflect a premorbid, non-pathological walking pattern given someone’s gait speed and a few individual characteristics that are easily collected.

Very few studies have investigated the prediction of neurotypical gait parameters. Most of these studies have developed methods to predict lower extremity joint angles and moments at different gait speeds^39–42^; which, compared to step length and peak anterior ground reaction force, are not often the focus of research or clinical interventions. While no prior work has predicted peak anterior ground reaction force observed during neurotypical gait, one study has attempted to predict the step lengths of neurotypical adults using age, height, body impedance, and muscle activity.^43^ Unfortunately, it is difficult to compare the performance of this model to the step length model reported here, as the model from Park, et al. was not validated on an independent dataset and our dataset does not have body impedance or muscle activity measures.

One could argue that age- and sex-based normative values for peak anterior ground reaction force^31^ and step length^32^ could be used to estimate the ideal magnitude of these parameters for an individual with gait dysfunction instead of employing more complicated prediction models like those developed here. However, age- and sex-based values for different gait parameters have limited generalizability to clinical populations as they are reported for gait speeds that are higher than typically observed in a clinical population. For example, in the “slow gait” condition tested in Öberg, et al. 1993, men between the ages of 50–59 walked at an average speed of 0.86 m/s. While this may be useful information for some, it does not allow for an accurate estimation of normative gait parameters for an individual that is not walking at a normative gait speed (e.g., a man who is 56 that is walking at 0.65 m/s following a stroke). This point is supported by the significant differences between the absolute error of the normative and predicted data observed here as well as the value of gait speed in the predictions indicated by the standardized beta weights (Tables 3 and 4). As such, the prediction models developed in this study make a unique contribution to this area of research by (1) providing a means to estimate the neurotypical magnitude of two clinically-relevant gait parameters at a wider range of speeds with greater accuracy than has been reported before and (2) validating these predictions on an independent data set.

One important implication of the prediction models developed in this study is that predicted peak anterior ground reaction force and step length values can be evaluated relative to the actual values in a population with gait dysfunction. These can then be used to determine the presence and magnitude of gait pattern deficits—without the need for demographically—and speed-matched neurotypical control data. This may significantly reduce the data collection burden for research studies and help inform clinical decision-making.

The ability to generate individualized reference values for peak anterior ground reaction force and step length provides a promising standard methodology to inform individualized gait biofeedback targets. To date, biofeedback targets for anterior ground reaction force and step length have largely been anchored to baseline walking patterns^18,22^. This is largely because targets anchored to baseline behavior are individualized and assumed to be achievable. However, it is possible that this approach generates target values that over- or underestimate the ideal target value. In cases where individuals have low baseline values for step length and anterior ground reaction force, this approach could mask additional capacity for change in movement patterns. We posit that using the values predicted from neurotypical gait behavior as a benchmark for the ideal target value would reduce the risk of masking someone’s capacity for change in studies of gait biofeedback. Though future work is necessary to determine the most appropriate way to implement the use of biofeedback targets based on neurotypical gait behavior to ensure the movement goals are still attainable, particularly if the magnitudes are much higher than baseline walking values.

Although we suggest the use of neurotypical gait behavior as a point of reference, we acknowledge that there is currently no consensus in the field on the “ideal” gait pattern for individuals within a given patient population. For example, people with Parkinson’s Disease generally benefit from feedback to increase their step lengths, but the exact step length magnitude that would optimize multiple aspects of walking (e.g., energy expenditure, dynamic balance, gait speed) for each person is poorly understood. With this work, we are assuming that the gait parameters of a neurotypical person with the same demographic characteristics walking at the same speed are a reasonable estimation of the “ideal” parameter values. While this logic may eventually be refuted with data as our understanding of optimal gait patterns in clinical populations with gait dysfunction evolve, there is currently no evidence to suggest that this approach is inherently flawed.

There are a few limitations to this study. First, the models are trained to predict a specific range of peak anterior ground reaction force and step length values and predictions outside of this range will be less accurate—as demonstrated by the larger RMSE of the step length predictions from the full independent validation dataset compared to that from the subset of data within the training set range. In addition to this, the models were trained on data with a specific range of anthropomorphic characteristics (age: 21–71 years; leg length: 0.65–0.91 m; mass: 45–95 kg), and the predictions may be less accurate for individuals who are outside these ranges. We also intentionally focused on predicting the gait metrics that are commonly studied and targeted with biofeedback in neurologic patient populations. However, other metrics may be of more interest or relevance in other diagnoses or gait pathologies (e.g., stance time asymmetry in individuals with lower extremity amputations^44^). Furthermore, we chose to use training data from participants that were not walking with the use of handrails which may limit the generalizability of these findings to clinical populations that often require the use of handrails for treadmill walking. This decision was made because the original training dataset only included four participants that used handrails while walking, which is not enough data to reliably evaluate the effect of handrail use on these gait parameters. Future work is necessary to assess the effect of using handrails on these gait parameter estimations. Finally, we did not test how well these equations can be generalized to overground walking though prior work suggests people walk with similar kinematic and kinetic metrics to those examined here during both treadmill and overground walking^45^.

In conclusion, we developed prediction models for peak anterior ground reaction force and step length that demonstrated strong agreement when validated on an independent dataset and better accuracy than normative data. The best prediction models included terms that captured gait speed and demographic characteristics (i.e., age, mass, leg length, sex). These models (and the associated calculation tables provided in the supplementary material S1) can be used to create individualized reference values of peak anterior ground reaction force and step length for use in gait rehabilitation research and clinical practice.

## Methods

### Training dataset

To train the prediction models, we used a publicly available dataset of 42 neurotypical adults walking on a treadmill at eight different speeds, ranging from 40 to 145% of their self-selected speed (mean of self-selected comfortable gait speeds: 1.25 ± 0.16 m/s; range of comfortable gait speeds: 0.89–1.54 m/s; total range of gait speeds: 0.36–2.23 m/s)^46^. At each speed, participants walked for 90 s, and data from the last 30 s of each trial were used in this analysis to allow the participant to adjust to each speed before recording their data. Kinematic data were recorded at 150 Hz and kinetic data at 300 Hz. To ensure accurate kinetic data, the data from four participants in the original dataset who used treadmill handrails while walking were removed. Another participant from this dataset was removed due to an aberrant offset in the force data. Therefore, we trained the models with data from a total of 37 neurotypical adults (age: 40 ± 17 years (21–71 years); leg length: 0.78 ± 0.07 m (0.65–0.91 m); mass: 67 ± 12 kg (45–95 kg) and sex (coded as male = 0 and female = 1)). For ten of the 37 participants, data from only six or seven (out of eight) trials were included in the analysis. Six of these participants were unable to complete the fastest walking speed. In the other four cases, the participants completed all walking trials, but we excluded the data from the trials in which the participants drifted across the midline of the instrumented treadmill (i.e., stepped on both the right and left force plates simultaneously). We were able to use the remaining data from these participants in our analysis because the statistical approach we employed (described below) is robust to missing data.

### Independent validation dataset

The independent dataset used to validate the final models consisted of 22 neurotypical adults (age: 61 ± 15 years (24–77 years); leg length: 0.78 ± 0.06 m (0.67–0.88 m); mass: 73 ± 18 kg (37–103 kg)) walking at their self-selected speed (0.48 to 1.29 m/s) for 2–5 min. These data were collected as part of another study, and the kinetic and kinematic data collection procedures have been previously reported^47^. Kinematic data were recorded at 100 Hz and kinetic at 1000 Hz. To keep the amount of data used for analysis consistent between the datasets, we extracted the first 90 s of each participant’s walking trial and used the last 30 s of that bin for analysis.

To account for the influence of other individual characteristics on peak anterior ground reaction force and step length, we extracted each participant’s age, leg length, sex, and mass. We defined leg length as the vertical distance from the greater trochanter marker to the lateral malleolus marker while the participant stood upright. Please see the previous publications^46,47^ for additional information about the data collection procedures.

### Normative peak anterior ground reaction force and step length data

To compare the performance of our model to previously published age- and sex-based norms, we extracted the normative peak anterior ground reaction force and step length values for each participant in the independent validation set. Normative values for peak anterior ground reaction force were obtained from Chao et al.^31^, which reports normative values for both men and women across a large age range. The normative peak anterior ground reaction force value for each participant was selected by the age and sex of each participant. Because these data were only reported for a narrow range of speeds (0.63–0.76 m/s), most of the normative estimates could not be speed-matched. Normative values for step length were obtained from Öberg, et al.^32^ because this paper reports normative data for men and women between the ages of 10–80 at three different gait speeds. The normative step length value for each participant was selected primarily based on age, sex, and the closest possible gait speed.

### Data processing

For both datasets, we analyzed the kinematic and kinetic data from the right lower extremity using MATLAB R2021a. Data from the left leg was not included in the analysis as the step length and anterior ground reaction force values on the right and left sides were highly correlated. For both datasets^46,47^, kinematic data were lowpass filtered with a cutoff frequency of 6 Hz^48^, while kinetic, at 20 Hz^47^. Vertical ground reaction forces were then used to identify kinetic gait events. Heel strike was identified at the point when vertical ground reaction force reached 100 N and toe-off at less than 100 N.

We defined peak anterior ground reaction force as the maximum anterior ground reaction force between heel strike and toe-off. The average peak anterior ground reaction force over each 30-s trial was used for all analyses. Step length was defined as the fore-aft difference between the right and left lateral malleoli markers at heel strike. The average step length over each 30-s trial was used for all analyses.

### Statistical analyses

We used linear mixed-effects models to determine if peak anterior ground reaction force and step length magnitudes were associated with speed (m/s), mass (kg), leg length (m), sex, and age (years). Given the known relationship between gait speed and both peak anterior ground force and step length^33^, the minimum model included a fixed effect for speed. Because visual inspection of the data indicated a non-linear relationship between speed and peak anterior ground reaction force and step length, we also considered models with a quadratic transformation of speed. In addition to these fixed effects, we included both a random intercept and a random slope for speed to account for repeated measures and between-subjects variability. The best subset model selection approach was used to identify the best model in the training data set. Best subset selection was performed by first comparing all possible models with the same number of variables and selecting the one with the highest adjusted R^2^ value to yield a candidate set of seven models^49^. Then models with different numbers of parameters were compared using the Akaike information criterion (AIC) and the model with the lowest AIC was selected as the final prediction model. This process was completed independently for peak anterior ground reaction force and step length.

We checked for linearity, multicollinearity, outliers, and influential points for both peak anterior ground reaction force and step length prediction models. The residuals vs fitted plots showed that, for the best models, there was no deviation from linearity. The variance inflation factors (VIF) for each of the coefficients were less than 5, indicating no major issues with collinearity. We identified one significantly influential point (one walking speed for a single participant) in the peak anterior ground reaction force model using Cook’s distance at a threshold of 0.11. We removed this influential point (0.3% of the total number of data points) and refit the final peak anterior ground reaction force model. Unlike for inference, the normality and homoscedasticity assumptions of linear mixed-effects modeling do not need to be met for prediction. For the same reason, we did not select the predictors based on p-values, as the best subset model selection process determines the model that provides the best prediction versus determining which predictors are most important^49^.

To understand the relative value of each variable in the prediction models, we determined the standardized beta weights for each variable. To do this, we first calculated the z-score for peak anterior ground reaction force, step length, and the continuous predictors to standardize them. Sex is a categorical variable, so it did not need to be standardized for this analysis. We refit the final models with the new standardized variables plus sex and used the betas from this analysis for comparison between predictors.

Next, we used an independent dataset (N = 22) to test the predictive ability of the peak anterior ground reaction force and step length models. We calculated predicted values for peak anterior ground reaction force and step length using the final models with only fixed-effects terms as the mean of the random term estimates from mixed-effects linear models is approximately zero^50^. We then calculated the R^2^ values and the root-mean-square error (RMSE) for the predicted versus actual peak anterior ground reaction force and step length. Because we found that some of the participants in the independent dataset walked with step lengths shorter than those represented in the training data, we also evaluated the R^2^ values and the RMSE of the predicted step lengths on a subset of the participants (n = 18) who’s actual step lengths were represented in the training dataset.

Finally, to evaluate the performance of our models to the performance of the normative data available we calculated the absolute error for both estimates (|predicted–actual| and |normative–actual|) of peak anterior ground reaction force and step length and compared these values with an unpaired t-test.

## Supplementary Information

Supplementary Information.

## Data Availability

The training data^30^ analyzed in this manuscript are publicly available in the PeerJ repository, https://peerj.com/articles/4640/. The independent validation dataset^31^ analyzed are available from the corresponding authors of the original manuscript upon reasonable request.

## References

[1] Highsmith MJ (2016). Gait training interventions for lower extremity amputees: A systematic literature review. Technol. Innov..

[2] Bland DC, Zampieri C, Damiano DL (2011). Effectiveness of physical therapy for improving gait and balance in individuals with traumatic brain injury: a systematic review. Brain Inj..

[3] Cadore EL, Rodríguez-Mañas L, Sinclair A, Izquierdo M (2013). Effects of different exercise interventions on risk of falls, gait ability, and balance in physically frail older adults: A systematic review. Rejuvenation Res..

[4] Abbruzzese G, Marchese R, Avanzino L, Pelosin E (2016). Rehabilitation for Parkinson’s disease: Current outlook and future challenges. Parkinson. Relat. Disord..

[5] Langhorne P, Bernhardt J, Kwakkel G (2011). Stroke rehabilitation. Lancet.

[6] Awad LN, Palmer JA, Pohlig RT, Binder-Macleod SA, Reisman DS (2015). Walking speed and step length asymmetry modify the energy cost of walking after stroke. Neurorehabil. Neural. Repair.

[7] Peterson DS, Mancini M, Fino PC, Horak F, Smulders K (2020). Speeding up gait in Parkinson’s disease. J. Parkinsons. Dis..

[8] Bower K (2019). Dynamic balance and instrumented gait variables are independent predictors of falls following stroke. J. Neuroeng. Rehabil..

[9] Thaut, M. H., Rice, R. R., Braun Janzen, T., Hurt-Thaut, C. P. & McIntosh, G. C. Rhythmic auditory stimulation for reduction of falls in Parkinson’s disease: A randomized controlled study. *Clin. Rehabil.* **33**, 34–43 (2019). 10.1177/0269215518788615 30033755

[10] Marques NR (2013). Association between energy cost of walking, muscle activation, and biomechanical parameters in older female fallers and non-fallers. Clin. Biomech. (Bristol, Avon).

[11] Hogue RE, McCandless S (1983). Genu recurvatum: Auditory biofeedback treatment for adult patients with stroke or head injuries. Arch. Phys. Med. Rehabil..

[12] Morris ME, Matyas TA, Bach TM, Goldie PA (1992). Electrogoniometric feedback: Its effect on genu recurvatum in stroke. Arch. Phys. Med. Rehabil..

[13] Finley JM, Bastian AJ (2017). Associations between foot placement asymmetries and metabolic cost of transport in hemiparetic gait. Neurorehabil. Neural. Repair.

[14] Ballaz L, Plamondon S, Lemay M (2010). Ankle range of motion is key to gait efficiency in adolescents with cerebral palsy. Clin. Biomech. (Bristol, Avon).

[15] van Gelder LMA, Barnes A, Wheat JS, Heller BW (2018). The use of biofeedback for gait retraining: A mapping review. Clin. Biomech. (Bristol, Avon).

[16] Hsu C-J, Kim J, Wu M (2018). Combined visual feedback with pelvic assistance force improves step length during treadmill walking in individuals with post-stroke hemiparesis. Annu. Int. Conf. IEEE Eng. Med. Biol. Soc..

[17] Padmanabhan P (2020). Persons post-stroke improve step length symmetry by walking asymmetrically. J. Neuroeng. Rehabil..

[18] Park S (2021). Using biofeedback to reduce step length asymmetry impairs dynamic balance in people poststroke. Neurorehabil. Neural. Repair.

[19] Sánchez N, Finley JM (2018). Individual differences in locomotor function predict the capacity to reduce asymmetry and modify the energetic cost of walking poststroke. Neurorehabil. Neural. Repair.

[20] Genthe K (2018). Effects of real-time gait biofeedback on paretic propulsion and gait biomechanics in individuals post-stroke. Top Stroke Rehabil..

[21] Liu J, Santucci V, Eicholtz S, Kesar TM (2021). Comparison of the effects of real-time propulsive force versus limb angle gait biofeedback on gait biomechanics. Gait Posture.

[22] Franz JR, Maletis M, Kram R (2014). Real-time feedback enhances forward propulsion during walking in old adults. Clin. Biomech. (Bristol, Avon).

[23] Browne MG, Franz JR (2018). More push from your push-off: Joint-level modifications to modulate propulsive forces in old age. PLoS ONE.

[24] Jellish J (2015). A system for real-time feedback to improve gait and posture in Parkinson’s disease. IEEE J. Biomed. Health Inform..

[25] Werner WG, Gentile AM (2010). Improving gait and promoting retention in individuals with Parkinson’s disease: A pilot study. J. Neurol..

[26] Spencer J, Wolf SL, Kesar TM (2021). Biofeedback for post-stroke gait retraining: A review of current evidence and future research directions in the context of emerging technologies. Front. Neurol..

[27] Nguyen TM (2020). Self-selected step length asymmetry is not explained by energy cost minimization in individuals with chronic stroke. J. NeuroEng. Rehabil..

[28] Leech, K. A. & Roemmich, R. T. Independent voluntary correction and savings in locomotor learning. *J. Exp. Biol.* **221**, jeb181826 (2018). 10.1242/jeb.181826 PMC6104817 29903840

[29] Roemmich RT, Leech KA, Gonzalez AJ, Bastian AJ (2019). Trading symmetry for energy cost during walking in healthy adults and persons poststroke. Neurorehabil. Neural. Repair..

[30] Schenck C, Kesar TM (2017). Effects of unilateral real-time biofeedback on propulsive forces during gait. J. Neuroeng. Rehabil..

[31] Chao EY, Laughman RK, Schneider E, Stauffer RN (1983). Normative data of knee joint motion and ground reaction forces in adult level walking. J. Biomech..

[32] Oberg T, Karsznia A, Oberg K (1993). Basic gait parameters: Reference data for normal subjects, 10–79 years of age. J. Rehabil. Res. Dev..

[33] Fukuchi CA, Fukuchi RK, Duarte M (2019). Effects of walking speed on gait biomechanics in healthy participants: A systematic review and meta-analysis. Syst. Rev..

[34] Hof AL (1996). Scaling gait data to body size. Gait Posture.

[35] JudgeRoy JO, Davis B, Ounpuu S (1996). Step length reductions in advanced age: The role of ankle and hip kinetics. J. Gerontol. A Biol. Sci. Med. Sci..

[36] Winter DA, Patla AE, Frank JS, Walt SE (1990). Biomechanical walking pattern changes in the fit and healthy elderly. Phys. Ther..

[37] McGibbon CA (2003). Toward a better understanding of gait changes with age and disablement: Neuromuscular adaptation. Exerc. Sport Sci. Rev..

[38] Franz JR (2016). The age-associated reduction in propulsive power generation in walking. Exerc. Sport Sci. Rev..

[39] Fukuchi CA, Duarte M (2019). A prediction method of speed-dependent walking patterns for healthy individuals. Gait Posture.

[40] Lelas JL, Merriman GJ, Riley PO, Kerrigan DC (2003). Predicting peak kinematic and kinetic parameters from gait speed. Gait Posture.

[41] Hanlon M, Anderson R (2006). Prediction methods to account for the effect of gait speed on lower limb angular kinematics. Gait Posture.

[42] Fukuchi CA, Fukuchi RK, Duarte M (2019). Test of two prediction methods for minimum and maximum values of gait kinematics and kinetics data over a range of speeds. Gait Posture.

[43] Park, J.-W., Baek, S.-H., Sung, J. H. & Kim, B.-J. Predictors of Step Length from Surface Electromyography and Body Impedance Analysis Parameters. *Sensors (Basel)* **22**, 5686 (2022). 10.3390/s22155686 PMC9371228 35957243

[44] Dingwell JB, Davis BL, Frazder DM (1996). Use of an instrumented treadmill for real-time gait symmetry evaluation and feedback in normal and trans-tibial amputee subjects. Prosthet. Orthot. Int..

[45] Lee, S. J. & Hidler, J. Biomechanics of overground vs. treadmill walking in healthy individuals. *J. Appl. Physiol.* **104**, 747–755 (2008). 10.1152/japplphysiol.01380.2006 18048582

[46] Fukuchi CA, Fukuchi RK, Duarte M (2018). A public dataset of overground and treadmill walking kinematics and kinetics in healthy individuals. PeerJ.

[47] Liu, C., McNitt-Gray, J. L. & Finley, J. M. Impairments in the mechanical effectiveness of reactive balance control strategies during walking in people post-stroke. 10.1101/2022.07.28.499225 (2022). 10.3389/fneur.2022.1032417 PMC9659909 36388197

[48] Winter, D. A. *Biomechanics and Motor Control of Human Movement*. (John Wiley & Sons, Inc., 2009). 10.1002/9780470549148.

[49] *An introduction to statistical learning: with applications in R*. (Springer, 2013).

[50] Skrondal A, Rabe-Hesketh S (2009). Prediction in multilevel generalized linear models. J. R. Stat. Soc. A. Stat. Soc..

